# A Comparative Study on the Structure and Quality of SLM and Cast AISI 316L Samples Subjected to WEDM Processing

**DOI:** 10.3390/ma15030701

**Published:** 2022-01-18

**Authors:** Magdalena Machno, Emilia Franczyk, Rafał Bogucki, Andrzej Matras, Wojciech Zębala

**Affiliations:** 1 Department of Rail Vehicles and Transport, Faculty of Mechanical, Cracow University of Technology, 31-155 Cracow, Poland; 2 Department of Production Engineering, Faculty of Mechanical, Cracow University of Technology, 31-155 Cracow, Poland; amatras@pk.edu.pl (A.M.); wojciech.zebala@pk.edu.pl (W.Z.); 3 Department of Materials Engineering, Faculty of Materials Engineering and Physics, Cracow University of Technology, 31-155 Cracow, Poland; rafal.bogucki@pk.edu.pl

**Keywords:** addictive manufacturing, selective laser melting, casting, AISI 316L, wire electrical discharge machining, surface roughness

## Abstract

Additive manufacturing technologies are increasingly used in the production of semi-finished workpieces intended for further processing. This entails the need to investigate the machinability and final properties of such products. Comparative research on wire electrical discharge machining (WEDM) processes performed with two kinds of AISI 316L stainless steel workpieces is presented in this paper. The first workpiece was made by selective laser melting (SLM), while the second one was casting. Both working materials were cut with current values ranging from 8 to 72 amps. A comparison of roughness, structure and chemical composition of machined surfaces was performed between the two kinds of specimens. For the SLM sample, parameters of the cutting process that provide relatively low surface roughness (*Ra* ≤ 10 µm) with the simultaneous maximization of the process efficiency were determined. It was found that in the case of applying high current values (72 amp.), more favorable properties of the treated surface were obtained for the SLM sample than for the cast one.

## 1. Introduction

Manufacturing technologies and materials engineering are closely related fields. Methods for producing specific elements are selected due to their shape, expected effects and properties of the processed material. In recent years, there has been an increasing demand for manufacturing technologies that enable the production of components with complex geometries, made of modern engineering materials such as titanium alloys, nickel-based superalloys and high-strength steels [1]. However, the physical, thermal and chemical properties of such materials make their machining difficult, especially when conventional methods are used [2,3,4]. An important aspect from an industrial point of view is the reduction of production costs whilst maintaining the high efficiency of the manufacturing process.

For the reasons stated above, additive manufacturing (AM) technologies are increasingly used [5,6]. Their growing popularity results from the possibilities they offer, such as the production of elements with very complex geometries (also internal) and shorter production times. The most popular AM technologies used for processing metals include powder bed fusion methods, which are divided into laser powder bed fusion (LPBF) and electron beam melting (EBM). Another method worth mentioning is direct laser deposition (DLD). A common method for production components of 316L stainless steel is LPBF, also known as selective laser melting (SLM) [7,8].

Unlike conventional, subtractive manufacturing processes, AM technologies are based on the gradual addition of material, layer by layer. A thin portion of metallic powder (below 150 µm) is spread on a dedicated platform and then fused by means of a laser beam [9]. An object is manufactured without the need for special tools and is characterized by high dimensional and high shape accuracy, relatively good surface quality and acceptable mechanical. AM processes automatically use three-dimensional (3D) models created in a computer-aided design/computer-aided manufacturing (CAD/CAM) environment, which makes them fast and allows the visualization of a final product [10]. Laser fusion of metal powders enables the production of advanced and lightweight structures that offer high strength and allow for weight reduction up to 60% compared to those produced by conventional means [6,11].

Possibilities provided by AM technologies make them more and more often used in various industries. For example, they are utilized for the production of turbine blades and fuel injector nozzles for aircraft engines and in the automotive industry—for prototyping and rapid manufacturing and repair of industrial hardware such as punches, dies and custom tooling. They are also used for producing injection molds equipped with internal cooling channels [5,12]. In medicine, 316L stainless steel processed by means of SLM is used for the rapid production of implants with complex geometries [13].

SLM technology is still a novelty. It was developed in the second half of the 1990s [14]. Currently, the structure and properties of SLM products are widely studied [9,15,16]. In paper [16], a sample made of 316L steel by means of SLM was subjected to microstructure analysis, Vickers hardness test, and wear test. The results were compared to those obtained for similar analyzes of a rolled sample made of the same material. The wear performance of SLM 316L was found to be better than in the case of a rolled sample. Research on the microstructure of the SLM sample showed that its upper surface consists mainly of cellular crystals, while the front surface and its section consists of columnar crystals. Furthermore, in [17], the microstructure and properties of SLM 316L samples were examined and compared to corresponding samples made by conventional methods. The research confirmed the unique structure of the former, which results from a large thermal gradient caused by rapid heating and then rapid solidification of the metallic powder. The yield strength of the SLM 316L steel sample was higher than that of the one obtained by conventional means.

In some cases, additional processing of SLM products is necessary in order to obtain desired surface roughness of manufactured parts. Values of *Ra* parameter for outer surfaces of such components are commonly above 10 or even 20 µm [15,18,19]. The authors of [20] presented a comprehensive literature review on surface roughness and morphology in the context of AM technology. They showed that their characterization is mainly based on 2D profile analysis and that the most frequently analyzed roughness parameter is the *Ra*.

The surface quality of a sample made of AlSi10Mg aluminum alloy using direct metal laser sintering and subjected to subsequent turning was analyzed in [21]. Roughness parameters *Ra* and *Rz* were found to depend mainly on the feed rate and corner radius of the tool. The lowest value of *Ra* (0.64 µm) was obtained for a feed rate of 0.058 mm/rev, cutting speed of 300 m/min, depth of cut of 1.0 mm, and corner radius of 0.4 mm. The lowest value of *Rz* (4.33 µm)—for a feed rate of 0.058 mm/rev, cutting speed of 200 m/min, depth of cut of 0.5 mm and corner radius of 0.2 mm. For a feed rate of 0.058, values of *Ra* and *Rz* parameters obtained for the DMLS sample were about 45% lower than that obtained for the cast sample. In [22], an attempt was made to reduce the roughness of the SLM sample made of AlSi10Mg by milling its surface. After an additional machining process, the roughness was reduced by twenty times. The authors managed to obtain a surface with a roughness of *Ra* = 0.14 µm and *Rz* = 1.1 µm. As a result of the optimization carried out, limit values of cutting speed (1100 mm/s) and feed rate (1300 mm/min) were determined for which *Ra* ≤ 0.2 µm and *Rz* ≤ 1.4 µm can be obtained. It was shown in [23] that after milling the SLM 316L sample with cutting speed of 60 m/min, even lower values of *Ra* (approx. 3.5 µm for the feed rate of 0.211 mm/rev) and *Rz* (approx. 14 µm for the feed rate of 0.211 mm/rev) could be achieved. Going further, the authors of [24] used wire electrical discharge polishing (WEDP) in order to improve the roughness of the 316L SLM sample. As an effect, a significant enhancement in the surface finish was achieved. The use of the WEDP process allowed to remove balling defects and the presence of unmelted or only partially melted particles. A decrease in porosity, as well as the removal of pits and cavities, was also observed. The *Sa* parameter was reduced to the value of 0.739 µm. The Austenite phase was found to be dominant before and after the WEDP process. However, the processed sample contained martensite, whose presence leads to an increase in material hardness.

The surface roughness of metallic parts and elements, including those produced with SLM, is often of great importance. Manufactured parts are often subjected to high-quality standards that provide their required service life. Adequate roughness of components external surfaces ensures their safe operation and proper alignment with other elements in the assembly. In the case of materials with low machinability, unconventional machining processes are used in order to improve their quality. One of the technologies that are suitable for processing this kind of materials and simultaneously allow to obtain sufficiently low roughness of the treated surface (*Ra* up to 1.0 µm for aluminum, 0.8 µm for brass, 0.7 µm for alloy steels [25]) is electrical discharge machining (EDM) [26,27]. Due to the high geometrical complexity of processed elements, a special type of EDM process, which is wire electrical discharge machining (WEDM), is sometimes used [28]. By applying complex relative movements (translational and angular) to a workpiece and a working electrode (wire), it is possible to process even very complex and small-scale objects [29]. Moreover, the use of low discharge energies (less than 5 mJ) can provide smooth surfaces (*Ra* = 0.5–2.5 µm) with slight thermal changes in the surface layer [30,31]. In addition, it is worth underlining that the WEDM process enables the machining of materials regardless of their physical properties. For this reason, this process was selected to perform experimental research instead of conventional methods such as milling or diamond turning, despite they also can provide low surface roughness, e.g., *Ra* = 0.23 µm [32] or *Ra* = 11.03 nm [33].

When analyzing the quality of the WEDM-treated product, it is important to examine the white layer formed on its surface. The thickness of the white layer depends mainly on the applied current value—the former increases with an increase in the latter. In [34], for the discharge current of 2–50 A, the average white layer thickness was in the range of 5–80 µm, respectively.

**Figure 1:**
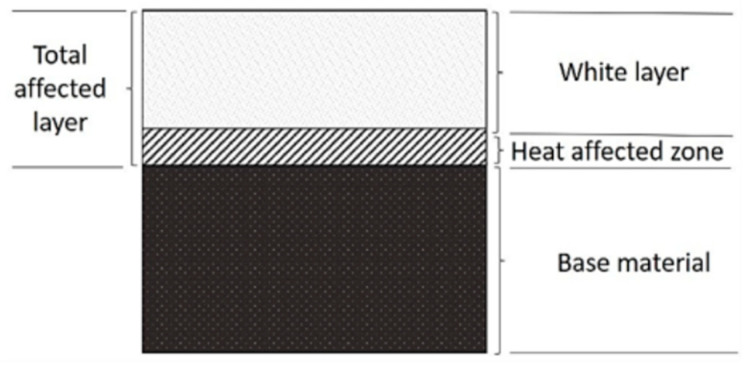
Scheme of the subsurface layers.

In Figure 1, a cross-section of the sample processed by means of WEDM is presented. What can be seen is the total affected layer, which is subdivided into [34,35]:

- white layer, made of melted material that was not thrown out to the surrounding medium and re-solidified,
- a heat-affected zone (HAZ, an area of increased hardness), characterized by a martensitic structure and a hardness greater than the original material.

There are surface irregularities such as pores, craters, and cracks present within the white layer [34]. It is also possible that a tempered, transition layer with a lower hardness than the original material is formed.

Compared to conventional machining methods, the EDM process is characterized by its low efficiency, which is a serious limitation [36]. Providing higher process speeds while maintaining sufficiently low surface roughness is a difficult task, in some cases requiring the use of two-stage operations. This is due to the fact that an increase in EDM efficiency stands in need for the application of high electrical discharge energy, which enhances the erosion effect and leads to the formation of a rugged surface with *Ra* reaching the value of 80 µm [37].

Results of the research performed on the condition of the surface layer formed after the WEDM process are presented in this paper. Samples made by means of both SLM and casting methods were compared. The use of the SLM product is a novelty, as this technique is still considered innovative and subjected to a lot of research. AISI 316L stainless steel was chosen as the material to produce specimens using both technologies. Current amplitude was adopted as a variable parameter of the WEDM process. The aim of the research was to investigate the condition of the surface layers formed after the WEDM process. An additional task was to select the process parameters ensuring surface roughness at the level of *Ra* ≤ 10 µm with a simultaneous maximum volumetric efficiency of the process.

## 2. Materials and Methods

### 2.1. Material

Austenitic stainless steel AISI 316L (X2CrNiMo17-12-2/1.4404) was used for research purposes. It was selected due to its wide application in various industries, including biomedical. Samples with dimensions of 4 mm × 5 mm × 20 mm were produced by two different methods, i.e., by casting and by SLM. According to F. Bartolomeu et al. [38], mechanical properties and subsequent wear behavior of the product are influenced by technologies used in the manufacturing process (Vickers hardness for the SLM sample—225 HV; for the cast sample—170 HV; tensile strength for the SLM sample—650 MPa; for the cast sample—450 Mpa; yield strength for the SLM sample—450 Mpa; for the cast sample—200 Mpa). The choice of these technologies resulted from the need to examine properties and surface quality of the sample made by SLM and subsequently cut with the use of WEDM process. Equally important was the comparison of the surface layer between said sample and the cast one, cut by means of the same process. Chemical composition of 316L metallic powder used in the SLM is shown in Table 1.

**Figure 2:**
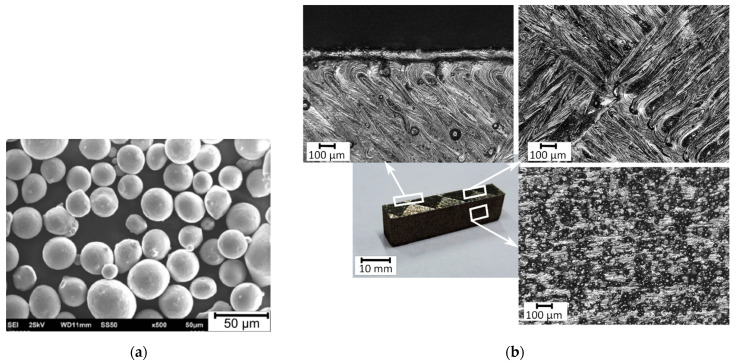
(**a**) Micro-image of the AISI 316L powder; (**b**) SLM printed sample and magnified views of its selected surfaces.

The SLM sample was made of metallic powder with a grain size in the range of 23–48 µm (Figure 2a) using the TRUMPF TruPrint 1000 3D Laser Metal Fusion printer (Ditzingen, Germany). Constant parameters of the SLM process are summarized in Table 2. Printed sample along with enlarged details of its selected side surfaces are shown in Figure 2b.

As for the cast sample, commercial alloy 316 L in the form of a rolled rod was used for the tests. In order to obtain a homogeneous austenite (without delta ferrite) structure, the samples were supersaturated at 1050 °C and cooled in water. As a result, the structure of homogeneous austenite with equiaxed grain and annealing twins was obtained.

### 2.2. Experiment Design

**Figure 3:**
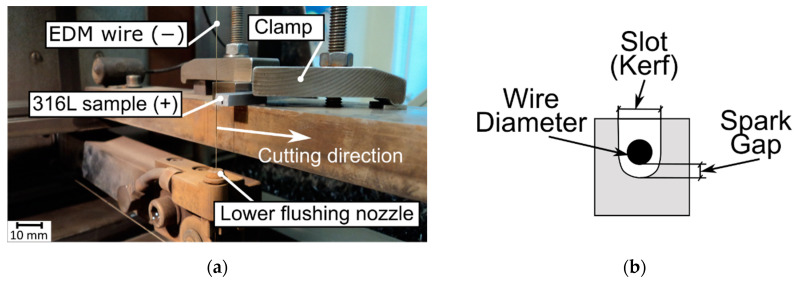
(**a**) Experimental test stand; (**b**) scheme of machining area.

**Figure 4:**
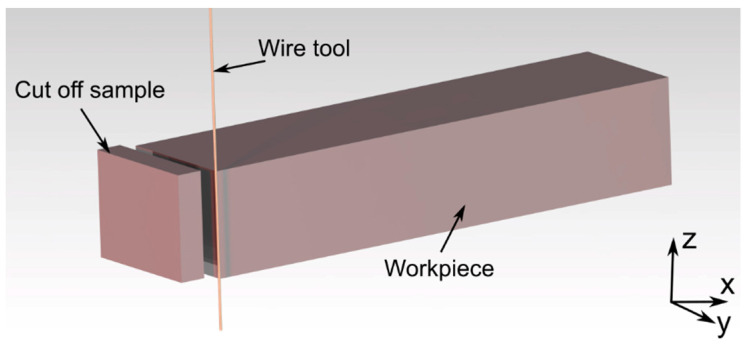
3D model of the WEDM process.

The WEDM process was carried out using BP95d electro-erosion cutting machine (Zakład Automatyki Przemysłowej B.P., Końskie, Poland). The sample mounted is shown in Figure 3a, while the outline of the machining area—in Figure 3b. The process consisted in cutting basic samples (made in SLM and casting processes) into pieces—cut off samples with dimensions of 1 mm × 5 mm × 4 mm. Length of the cut was 5 mm. In the case of SLM sample, cutting process was performed in the direction perpendicular to its layers. Exact method of cutting the samples is shown in Figure 4.

The research was aimed at examining surface layer formed after cutting the samples by means of WEDM process. For this reason, current amplitude was adopted as the variable input process parameter. As it is known, it has the greatest impact on the structure of machined surface layer. Tests were carried out for current values in the range of 8 ÷ 72 A and with a step of 16 A. Three repetitions were made for each test setup. Test conditions, as well as the cutting process parameters, are listed in Table 3.

In order to determine the roughness of processed surface, appropriate measurements of the *Ra* parameter was made. For this purpose, a Taysurf Intra 50 profile measuring tool (Taylor Hobson, Leicester, UK) equipped with a measuring tip with a rounding radius of 2 µm was used. The measurements were taken in a direction parallel to the cutting direction. A single measurement was carried out over a 1.0 mm long section and with a speed of 1 mm/s. Three repetitions were performed on each surface. Presented values of roughness parameters are calculated means.

The SEM—EDS (Scanning Electron Microscopy—Energy Dispersive Spectroscopy) technique was used in order to examine microstructural changes in the heat-affected zone. A JOEL JSM5510LV microscope (Tokyo, Japan) was utilized for this purpose. Test samples were polished with the use of diamond paste in a direction perpendicular to the cutting direction and then electrolytically etched using a chromium reagent CrO_3_. EDS was used in order to determine chemical composition in the micro-areas of the test samples.

**Figure 5:**
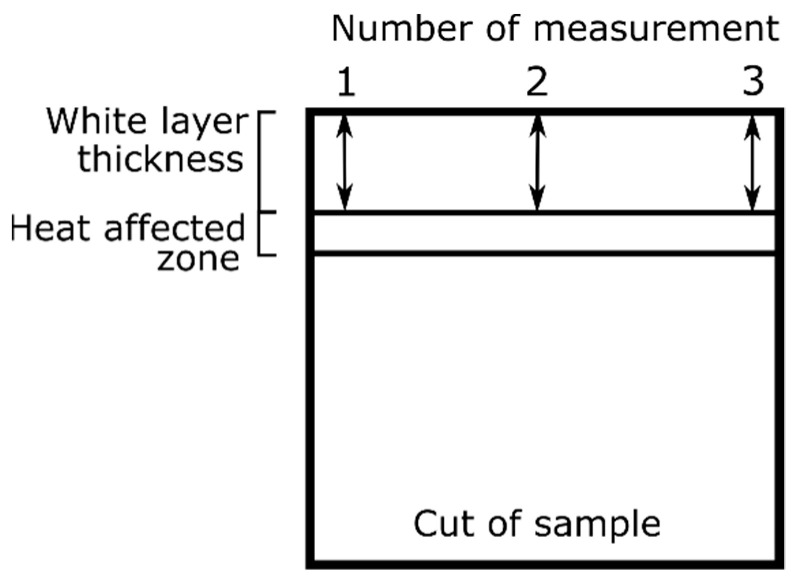
Scheme of layer measurement method.

Thicknesses of the white layer and the heat-affected zones were measured by means of a scanning microscope. Measurement of white layer thickness was performed on its two sides and in the middle, according to the scheme presented in Figure 5. In total, three measurements were made for each layer, and the mean value was calculated. In the case of heat-affected zone, the measurements were performed in areas where the visibility of the layer was sufficient.

As part of the research on the WEDM process, values of volumetric cutting rate (*V_w_*) were determined for both types of samples according to the following formula:

*V_w_* [m^3^/s] = (*m*_1_ − *m*_2_)/(*ρ* × *t_m_*), (1)

where: *m*_1_—weight of workpiece (before cutting) [kg], *m*_2_—weight of workpiece and the cut off sample (after cutting) [kg], *ρ*—density of AISI 316L stainless steel [kg/m^3^], *t_m_*—cutting time [s].

Statistical analysis of measured data (*Ra*, *V_w_* and white layer thickness) was performed using the One-Way Analysis of Variance (ANOVA) method. Analyses results are presented in Table 4, Table 5, Table 6, Table 7, Table 8 and Table 9, respectively. In each of them, *DF* is degrees of freedom, *Seq SS* is sums of squares, *Adj SS* is the adjusted sums of squares, and *Adj MS* is the adjusted means squares.

## 3. Results and Discussion

Condition of the surface and subsurface layers of elements produced by means of electro-erosion machining significantly affects their subsequent operation. When analyzing it, one should consider the effects of thermal mechanisms, i.e., heating, melting and evaporation of the processed material. Processes of energy and mass transfer taking place in the inter-electrode gap cause alterations in specific areas of the workpiece. The surface roughness analysis is an essential study, but it is also important to examine the layers changed as a result of melting and re-solidification of the material due to expansion of the high-temperature zone. The depth of a thermally changed layer depends, inter alia, on the processing conditions and on the type and properties of processed material [3].

### 3.1. Analysis of the Surface Structure for Different Current Amplitudes

**Figure 6:**
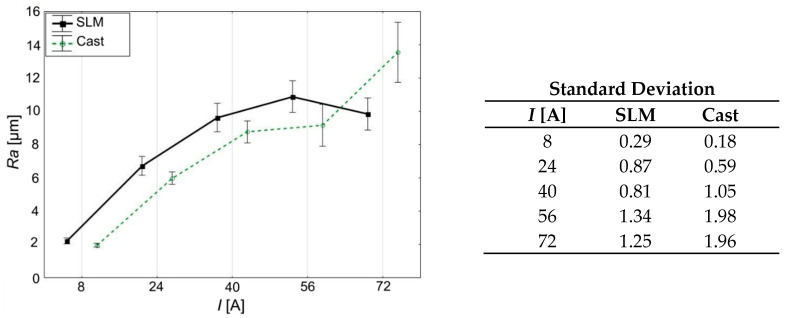
Relationship between current amplitude (*I*) and surface roughness *(Ra*).

The surface machined by the WEDM process contains a series of mutually overlapping erosion craters with a shape similar to spherical cups, which together form its geometric structure. As the current value increases, an increase in *Ra* is also observed (Figure 6, Table 4 and Table 5). The reason is the fact that the higher the current value in a process is, the greater is the energy of a single discharge. This causes larger amounts of material to melt and vaporize and thus creates bigger erosion craters.

For current amplitudes in a range of 4 ÷ 56 A, no significant differences in surface roughness were found between cast and SLM samples. Similar values of the *Ra* prove that the differences in their structure do not significantly affect their roughness after this kind of machining. Thus, for the tested steel and aforementioned range of currents, values of WEDM process parameters similar to those used for castings can be used while machining SLM workpieces.

For the cast sample, increasing the current amplitude from 56 to 72 A resulted in an increase in *Ra* from 9 to 14 µm, which is about 30% of the initial value. In the case of the SLM sample, increasing the current to 72 A did not deteriorate the roughness of the cut surface. *Ra* value was, in this case, similar to the values obtained for *I* = 40 A and *I* = 56 A, i.e., at a level of about 10 µm. Comparison of SLM and cast samples, with both cut with a current of *I* = 72 A, indicates a 25% lower *Ra* value of the first one.

**Figure 7:**
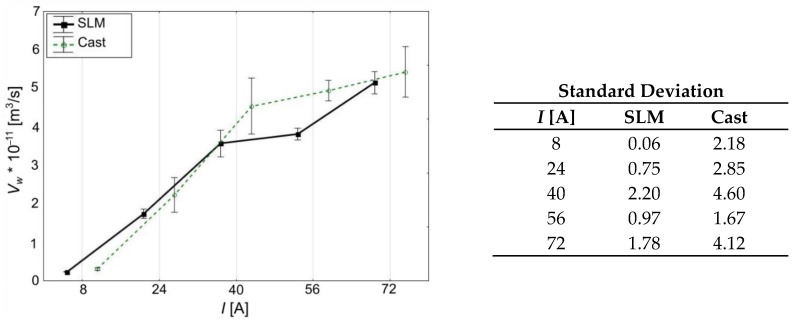
Relationship between current amplitude (*I*) and volumetric cutting rate (*V_w_*), 95% confidence intervals.

Analysis of the results in terms of WEDM process volumetric efficiency (*V_w_*) correctly indicates an increase in this parameter with an increase in the applied current amplitude (Figure 7, Table 6 and Table 7). It should be noted that during the processing of the SLM workpiece with the current value of *I* = 72 A, maximum volumetric efficiency of the process is obtained (about 5 × 10^−11^ m^3^/s), and still the surface roughness of *Ra* ≤ 10 µm is maintained. Surface roughness analysis did not explain why the roughness obtained when using 72 A current is at a similar level as in the cases of 40 A and 56 A. Either way, the values obtained suggest that the use of SLM technology has an effect on the quality of the final surface, which is a product of WEDM post-processing.

Observations described in the two paragraphs above are important in assessing potential of using SLM method for the production of semi-finished products as well as of using the WEDM process for their subsequent processing.

**Figure 8:**
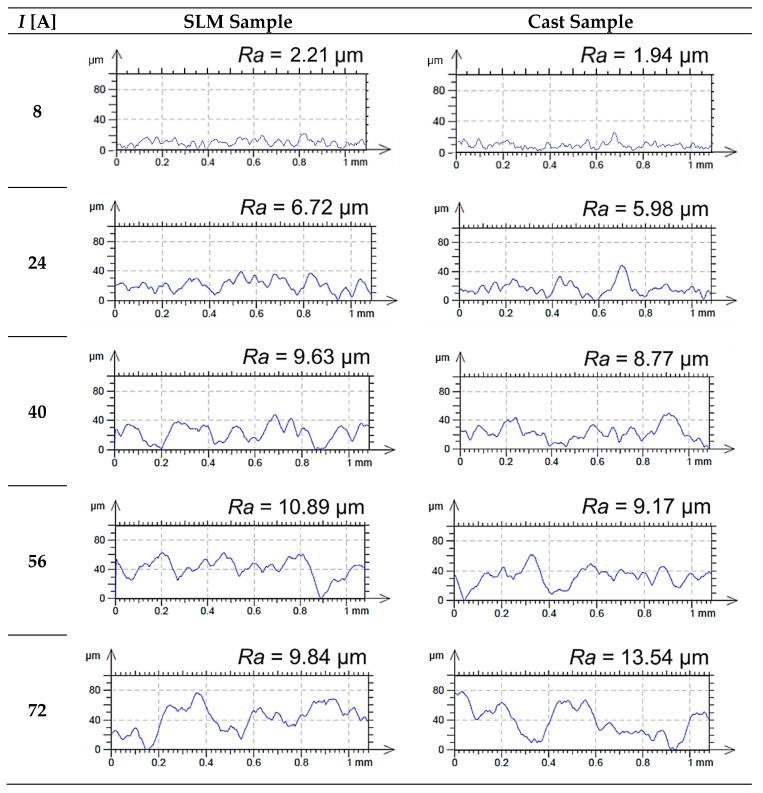
2D profiles of machined surfaces of SLM and cast samples for the analyzed values of current amplitude (*I*).

In comparing the profiles of machined surfaces, it can be observed that for a given current value, they are similar, regardless of the sample type (Figure 8).

**Figure 9:**
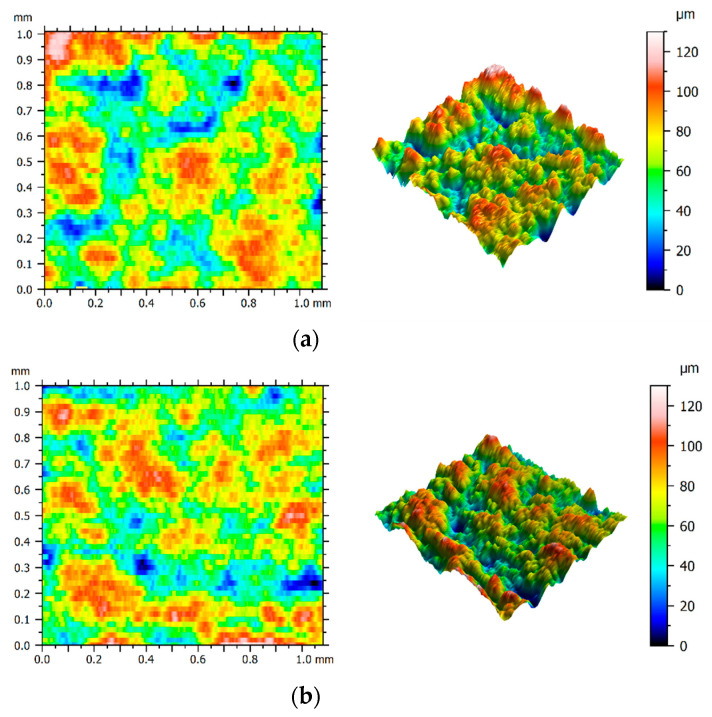
Surface texture of the SLM sample for current amplitude of (**a**) 56 A; (**b**) 72 A.

**Figure 10:**
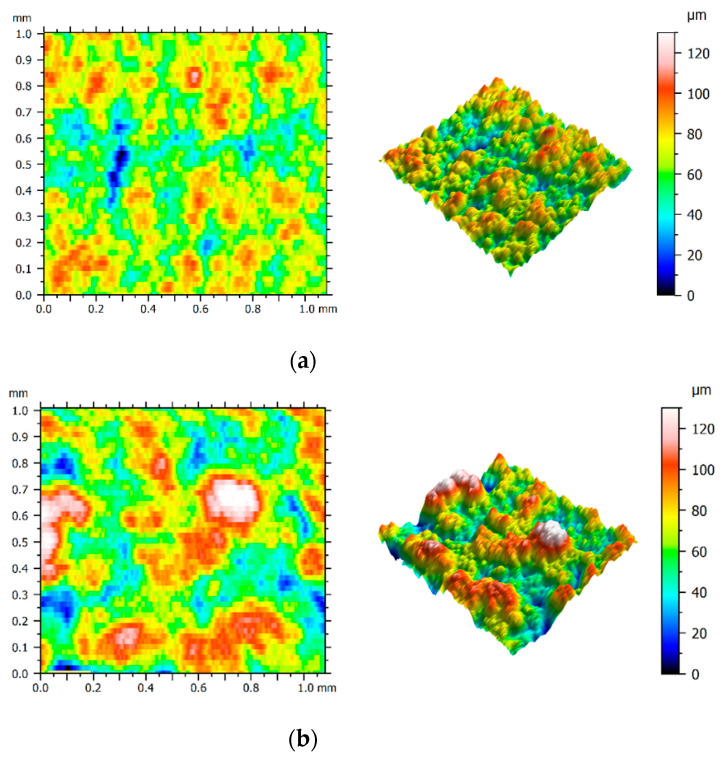
Surface texture of the cast sample for current amplitude of: (**a**) 56 A; (**b**) 72 A.

In the case of the SLM sample, the *Ra* parameter values obtained for the current of *I* = 72 A were at the level of those obtained for *I* = 56 A. Three-dimensional representations of the surfaces made using these two current values are presented in Figure 9a,b. On the basis of the attached figures, it can be concluded that despite a significant difference in the applied current (16 A), both surfaces have a similar range of roughness. For comparison purposes, structures of the cast sample surfaces produced using the current of *I* = 56 A and *I* = 72 A are presented in Figure 10a,b, respectively.

Surface roughness analysis proved that the use of SLM technology strongly influences the final properties of the surface treated with the WEDM process. In order to investigate this relationship in more detail, SEM and EDS analyzes of the machined layer were performed.

### 3.2. Microstructure of Cast and SLM Samples

**Figure 11:**
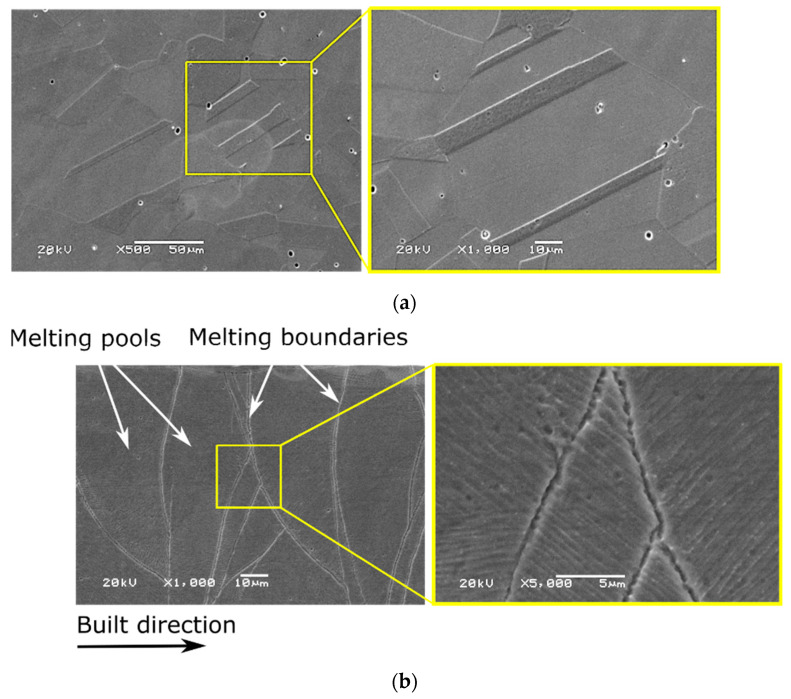
SEM images of the original structure of the samples: (**a**) cast; (**b**) SLM.

SEM pictures presenting the initial structures of the cast and the SLM samples are shown in Figure 11a,b, respectively. Both types of analyzed samples, regardless of their manufacturing process, had an austenitic structure. In the case of 316L solid steel (Figure 11a), the microstructure consists of equiaxed austenite grains with visible glow (recrystallization) twins, which were formed after the annealing process [39]. In the case of the SLM sample, cellular microstructures related to rapidly solidified austenitic stainless steel are observed [39], which indicates a directional nature of heat propagation during the manufacturing process (Figure 11b). Melting pools and melting boundaries appear within the structure of the SLM sample, and the microstructure is characterized as non-homogeneous [40].

The use of SLM resulted in obtaining a characteristic structure created by epitaxial growth of austenite grains, which was also observed in the works [41,42]. The elongated form of grains observed results from the action of a laser beam [43,44]. A dendritic system visible within austenite grains, Figure 11b, was described in [43]. A positive influence of SLM on the size of grains was found in [45]. It is related to the fast crystallization process taking place after material remelting. The use of the Jeffries method for grain size analysis revealed a slight difference in the mean diameter (*d_av_*) of the austenite grain. The *d_av_* value was 54 µm for SLM samples, while for solid steel, it was 49 µm. Such a slight difference is probably due to the low laser power of 90 W.

### 3.3. Surface Layer Analysis

For the needs of the surface layer analysis, detailed SEM images were taken. They present top views of the treated surfaces as well as cross-sections of the machined samples.

**Figure 12:**
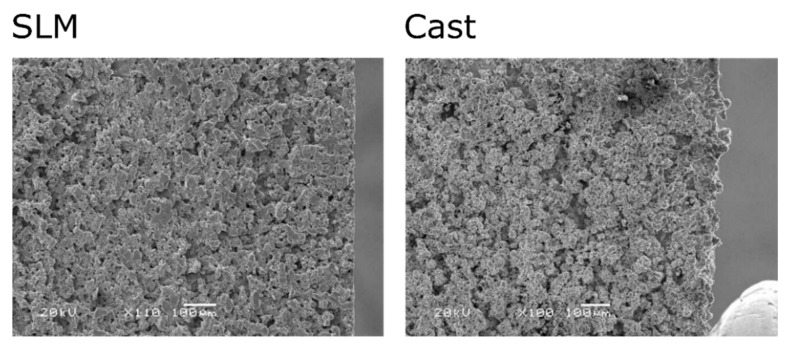
Top view the machined surface of SLM and cast samples for *I* = 72 A.

When analyzing the top views of both samples, it can be clearly seen the white layer structure for the cast sample is rougher. This is clearly visible in the SEM photos for the case of using a current of 72 A (Figure 12). It can also be seen that in the case of a casting surface, irregularities extend beyond its edge. On the other hand, the edge of the SLM sample is straight, which indicates its more condensed structure and also proves better shape accuracy.

**Figure 13:**
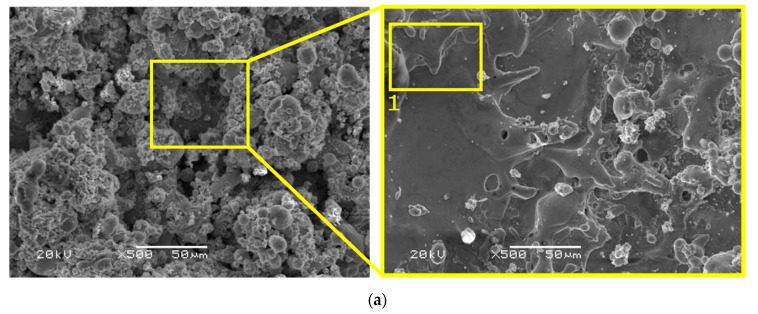

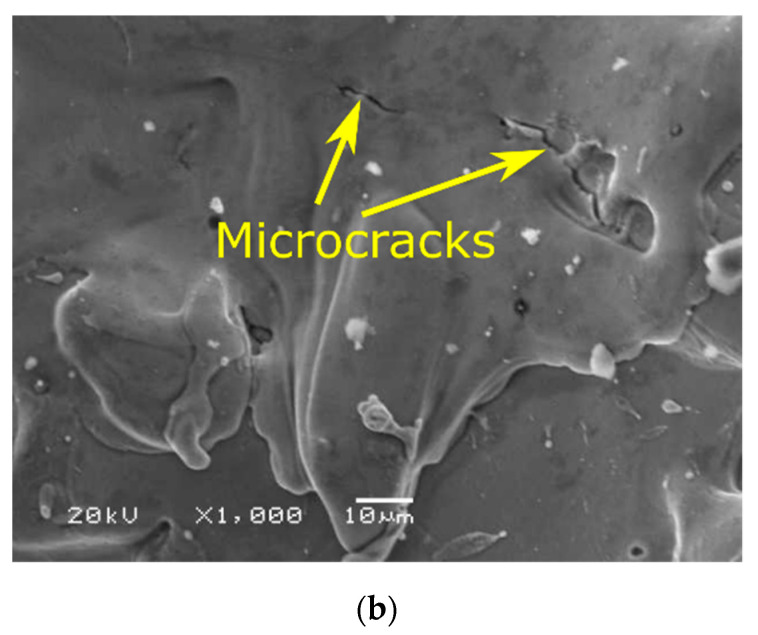
SEM images of top view for cast samples (**a**); magnification of point 1 (**b**); for using *I* = 72 A.

**Figure 14:**
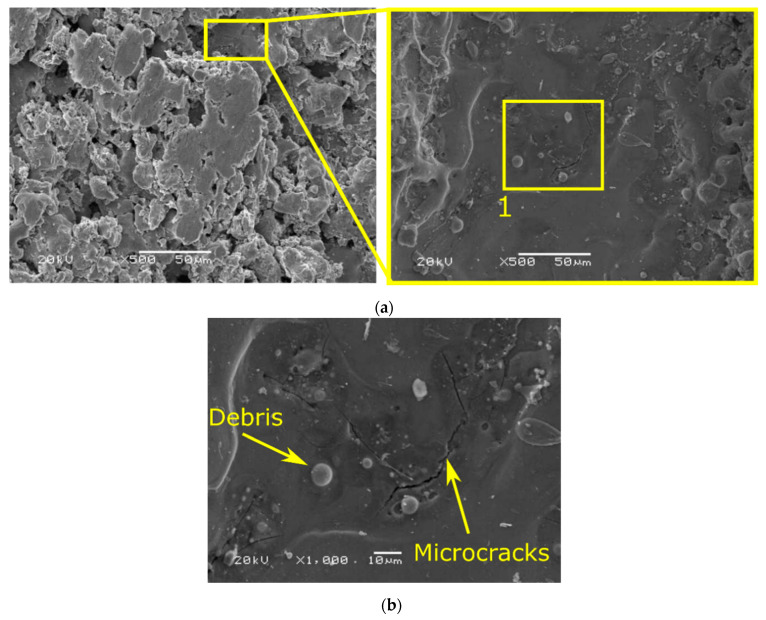
SEM images of top view for SLM samples (**a**); magnification of point 1 (**b**); for using *I* = 72 A.

Further analysis allows observing defects characteristic for EDM treatment, such as microcracks or the presence of debris (Figure 13 and Figure 14). When comparing the samples, it can be seen that a greater number of microcracks appear on the surface of the SLM sample, which is due to its greater hardness. According to [38], components made of 316L steel by means of SLM have higher hardness than castings. As a result, they are also more prone to cracking and chipping processes.

**Figure 15:**
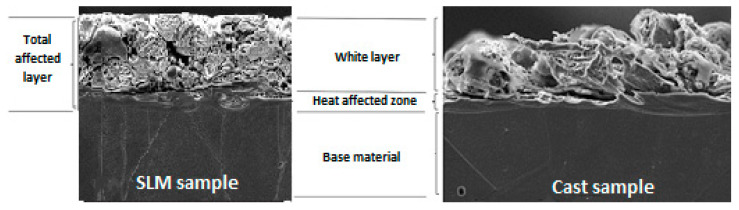
Cross-sections and schemes of the subsurface layers for SLM and cast samples.

Next, the treated surface was examined through its cross-section analysis. In Figure 15, exemplary images of samples cross-sections are presented together with an explanatory scheme. As can be seen, regardless of the type of sample, the following layers differ from each other. The white layer is a porous, torn zone that is less condensed for a cast sample. The heat-affected zone is consistent and homogeneous, and the base material is a zone where no structural changes occur. It is also possible that a tempered, transition layer with a lower hardness than the original material is formed.

**Figure 16:**
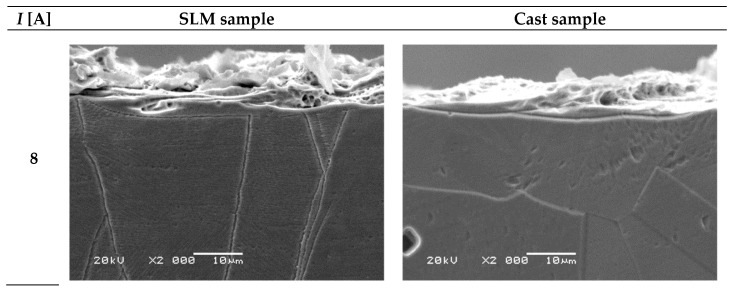

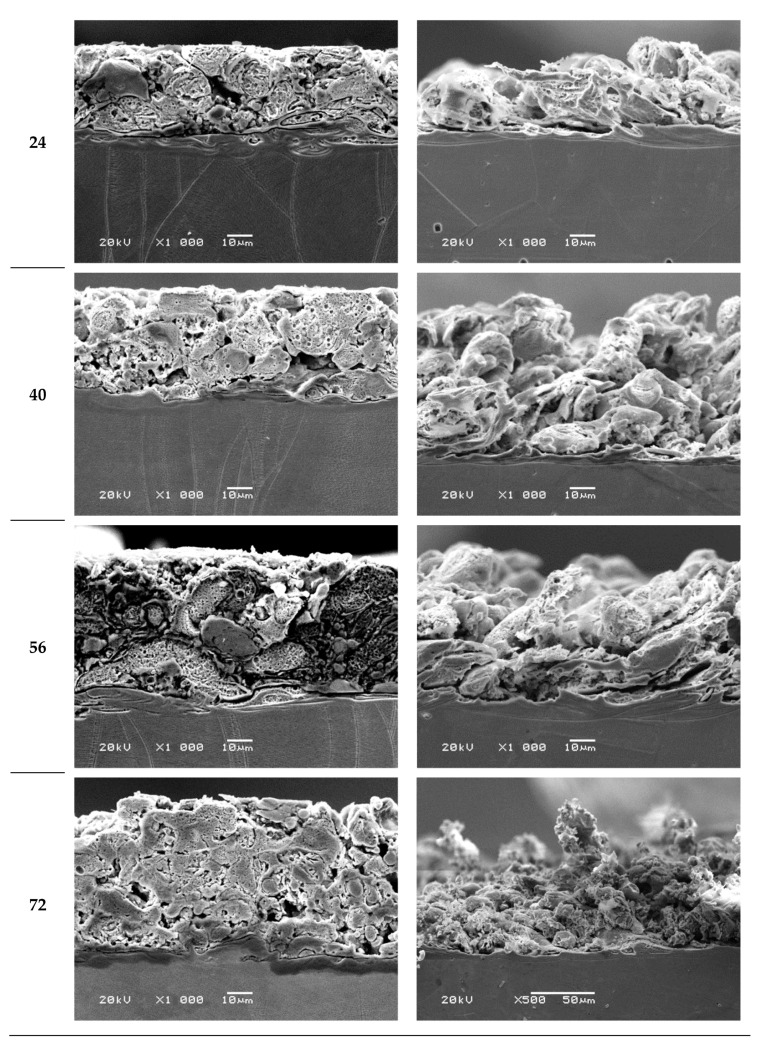
SEM images of white layer microstructure for the SLM and the cast samples for various current values (*I*).

Figure 16 presents SEM pictures of specific microsections with a visible white layer. Despite a similar, austenitic structure, it can be observed that for a given current value, its structure differs depending on the type of sample. It is also clear that WEDM post-processing significantly affects the form and size of the white layer.

White layer produced on the cast sample has a highly irregular structure with countless depressions and elevations that increase its roughness. Such a condition results from the presence of numerous overlapping erosion craters. In this case, a high current value (*I* = 72 A) caused significant deterioration of the surface. On the other hand, the SLM sample has a denser structure with numerous pores and areas of re-solidified material. This is caused by re-exposing the workpiece material to a high temperature caused by electrical discharge and forcing the material to melt and vaporize. During the SLM process, metallic powder has already been subjected to a temperature exceeding its melting point. Because of a dense structure of SLM samples, for a current range from *I* = 40 A to *I* = 72 A, *Ra* parameter had similar values. This observation proves that the technology of producing semi-finished products is important in the context of further WEDM process results.

Additionally, differences in the microstructure of both samples allow concluding that they are influenced by the nature of heat penetration into the material. In the case of SLM samples, it can be seen that the higher the current in the cutting process is, the more condensed is the white layer. This explains the phenomenon that surface roughness stops increasing above a certain current value, which in this case is 40 A. When a higher value of current is used (up to 72 A in this case), it is possible to achieve relatively smaller surface roughness with a simultaneous maximum material removal rate of WEDM. According to this information, the use of WEDM as an additional process aimed at improving surface quality has great potential.

**Figure 17:**
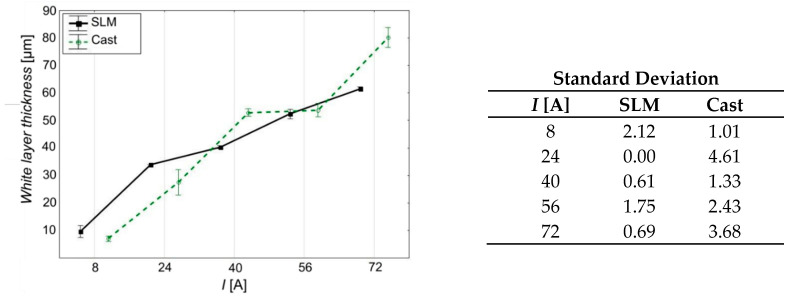
Relationship between current amplitude (*I*) and white layer thickness.

Based on the white layer thickness measurement results presented in Figure 17 and analyzed in Table 8 and Table 9, it was found that as the current value increases, its thickness also increases, regardless of the type of material. This dependence is due to the fact that an increase in the current brings more energy to every single electrical discharge, thus causing an increase in the amount of heat acting on the treated surface. With a greater amount of heat, changes in the material structure occur to a greater extent. As for the cast sample, with a current value of *I* = 72 A, a sudden increase in this value (over 20% compared to that achieved with a current of 56 A) is observed. In the case of SLM samples, the thickness of the white layer gradually increases over the entire range of currents used.

It is known that in terms of surface roughness, a thin white layer is preferable to a thick one [46]. An increase in the amount of eroded material also causes an increase in the thickness of the material layer not removed from the discharge crater, which solidifies and forms a white layer. As the defects present in the white layer are strongly related to its thickness, less thickness provides less surface roughness. This proves that also in this context WEDM process with the use of high current values gives better results when processing an SLM sample rather than a casting.

**Figure 18:**
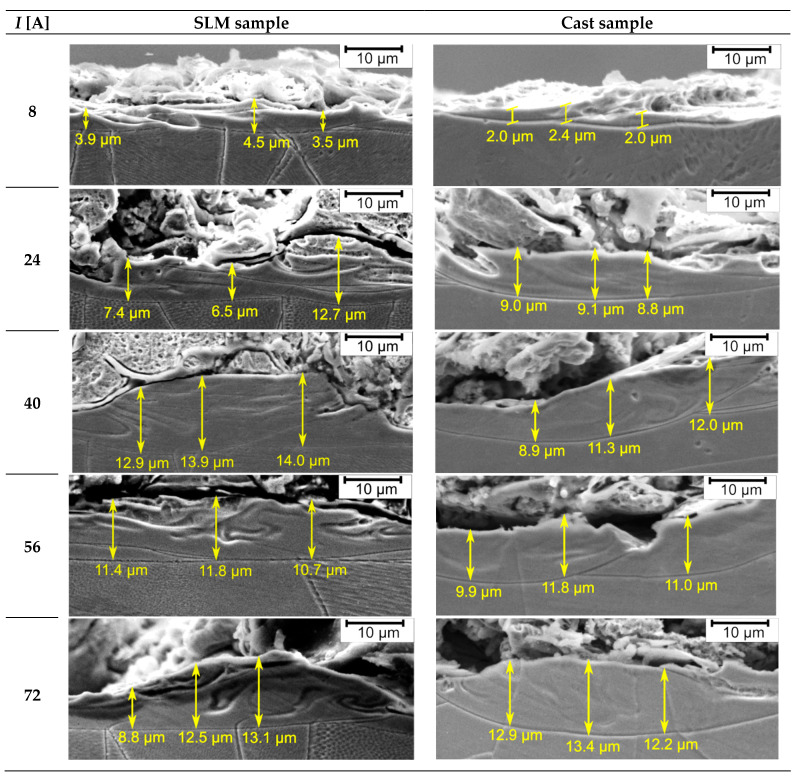
SEM images of microstructure of heat-affected zone for the SLM and the cast samples for various current values (*I*).

Performed research also showed that a heat-affected zone (HAZ) is formed under the white layer and that its thickness is in the range of 2.0 ÷ 14.0 µm (Figure 18). For a fixed current value, HAZ thickness is similar for both tested materials. Because the white layer was irregularly shaped, presented values of HAZ thickness are illustrative only. Therefore, their means have not been determined. It can be seen that the white layer structure is oriented and that its direction corresponds to the direction of wire electrode movement.

The lowest values of HAZ thickness (about 2.0 ÷ 4.5 µm) were obtained for current of *I* = 8 A, regardless of sample type. This is due to the fact that a relatively small amount of thermal energy is delivered to the processed material and, as a result, the range of thermal changes inside it is smaller. For the remaining current values (*I* = 24 ÷ 72 A), the thickness of HAZ is approximately 11 µm.

### 3.4. EDS Analysis for Cast and SLM Samples

**Figure 19:**
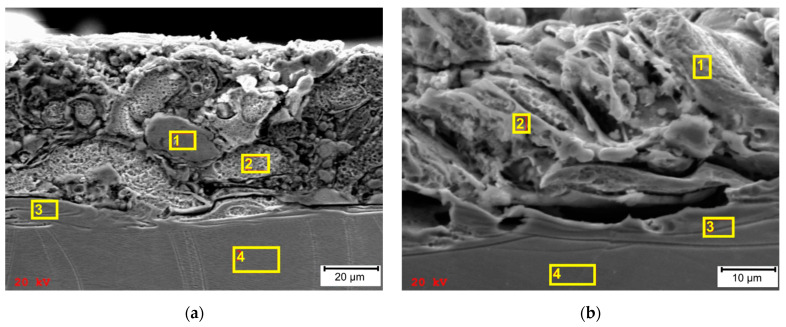
SEM images of total heat-affected zone with designation of specific EDS surface analysis areas: (**a**) SLM sample; (**b**) cast sample.

Selected areas of metallographic specimens were subjected to quantitative analysis with the use of EDS (Figure 19). In the surface layers of both types of samples, there are mainly constituent elements of 316L stainless steel such as chromium, iron and nickel (Table 1). Analysis of the unchanged areas of material also showed a similar chemical composition between the casting and SLM samples (Table 10).

Oxygen was found to be present in analyzed areas of samples processed by WEDM. Specific conditions that occur during such a process, i.e., a high temperature within the inter-electrode gap (6000 ÷ 10,000 °K) and the use of demineralized water as a working fluid, caused the formation of an oxide layer. Some amount of oxygen is present in zones 3 and 4 of the cast sample (1.00 [wt. %] and 0.88 [wt.%], respectively). In the case of the SLM sample, oxygen content in individual areas ranged from 0.80 to 1.75 [wt. %]. The research by Sun et al. [39] indicates that the occurrence of oxygen results from the presence of trace oxygen in the processing area, which explains the small amount of oxygen found in zones 3 and 4. Greater amounts of oxygen can be found in the white layers of both samples. In the case of casting, it is 2.53 [wt.%] in zone no.1 and 11.47 [wt.%] in zone no.2. In the case of the SLM sample, it is 1.75 [wt.%] in zone no.2 (Table 10). This supports the conclusion that the presence of oxygen in the white layer results from the high-temperature phenomena taking place during the WEDM process, in which demineralized water is used as dielectric fluid. The presented results confirm the phenomenon of oxidation of the surface layer during the EDM process. Due to the low measuring accuracy of the EDS system in the case of light elements, the remaining results are of qualitative nature. Chemical composition, especially the presence of heavy elements such as Mo, may influence the flow of heat into the material [47,48].

## 4. Conclusions

The presented paper includes the results of a comparative analysis of samples made of AISI 316L steel. Specimens were made by means of both SLM and casting processes and then cut with the use of WEDM. Differences in surface roughness and characteristics of thermally changed layers were found between the specimens.

Significant differences in the *Ra* roughness parameter were observed between the samples for high current values. In the range of *I* = 56 ÷ 72 A, the casting is much more sensitive to increase in this parameter. Changing the current from 56 to 72 A causes an increase in *Ra* value by 30%, up to about 14 µm. On the contrary, the SLM maintains the *Ra* at a constant level of about 10 µm. For current of *I* = 72 A, *Ra* values for the SLM sample were about 25% lower compared to the cast one. It indicates that relatively good surface quality, as well as improved process efficiency, can be obtained while processing material made by means of SLM. Such an effect is not achievable for the cast sample, which proves the benefit emerging from using the SLM method.

To sum up, the research proved that the type of manufacturing technology of semi-finished products significantly affects the results of following WEDM post-processing in terms of surface roughness. Furthermore, significant differences have been found in white layer structure between both types of samples subjected to WEDM. A fact of great importance is that for the SLM sample, it was possible to achieve a relatively low value of the Ra roughness parameter (about 10 µm) together with the maximum value of material removal rate.

It can be concluded that, as a method of producing semi-finished products, SLM has advantages that make it competitive to conventional methods such as casting. However, this process is relatively expensive and time-consuming, which is its greatest limitation.

Further experimental work should include analysis of the influence of other WEDM process parameters, e.g., pulse on time, pulse off time and open voltage on the properties of surface layers of cast and SLM samples. It is also advisable to optimize the process to increase its efficiency and to improve the surface quality of manufactured objects. It is also necessary to further analyze the effects of heat on the processed material.

## Figures and Tables

**Table 1:** Chemical composition of AISI 316L powder [%].

C	Si	Mn	Cr	Ni	Mo	Fe
<0.03	<1.0	<2.0	16.0–18.0	11.0–14.0	2.0–3.0	Ballance

**Table 2:** SLM process parameters.

Parameter	Value
Laser power, [W]	90
Layer thickness, [μm]	20
Inert gas speed, [m/s]	2.5
Laser speed, [mm/s]	500
Coater return speed, [mm/s]	250
Coater work speed, [mm/s]	80
Oxygen level, [%]	0.3

**Table 3:** WEDM process parameters and machining conditions.

Machining Parameter	Value/Characteristic
Pulse on time, *t_on_* [µs]	10
Pulse off time, *t_off_* [µs]	350
The interelectrode gap size, *S* [mm]	0.28
Wire feed rate, *v_f_* [mm/s]	10
Current amplitude, *I* [A]	8; 24; 40; 56; 72
Material of wire tool electrode	Brass
Wire tool diameter, [mm]	0.25
Working fluid	Demineralized water with electrical conductivity 89.5 µS/cm
Temperature of working fluid, *T* [°K]	~294

**Table 4:** ANOVA—*Ra* for SLM sample.

Source	DF	Adj SS	Adj MS	F-Value	*p*-Value
*I* [A]	4	147.569	36.8923	291.59	<0.001
Error	10	1.265	0.1265		
Total	14	148.835			

**Table 5:** ANOVA—*Ra* for casting.

Source	DF	Adj SS	Adj MS	F-Value	*p*-Value
*I* [A]	4	210.447	52.6116	187.37	<0.001
Error	10	2.808	0.2808		
Total	14	213.254			

**Table 6:** ANOVA—*V_W_* for SLM sample.

Source	DF	Adj SS	Adj MS	F-Value	*p*-Value
*I* [A]	4	59.0156	14.7539	773.53	<0.001
Error	15	0.2861	0.0191		
Total	19	59.3017			

**Table 7:** ANOVA—*V_W_* for casting.

Source	DF	Adj SS	Adj MS	F-Value	*p*-Value
*I* [A]	4	74.573	18.6433	189.68	<0.001
Error	15	1.474	0.0983		
Total	19	76.047			

**Table 8:** ANOVA—white layer thickness for SLM sample.

Source	DF	Adj SS	Adj MS	F-Value	*p*-Value
*I* [A]	4	4738.92	1184.73	704.92	<0.001
Error	10	16.81	1.68		
Total	14	4755.73			

**Table 9:** ANOVA—white layer thickness for casting.

Source	DF	Adj SS	Adj MS	F-Value	*p*-Value
*I* [A]	4	12,734	3183.4	26.90	<0.001
Error	10	1183	118.3		
Total	14	13,917			

**Table 10:** Chemical composition in the specific areas of the SLM sample and cast.

Main Elements	SLM									Cast						
	Zone 1		Zone 2		Zone 3		Zone 4		Zone 1		Zone 2		Zone 3		Zone 4	
	at. [%]	wt. [%]	at. [%]	wt. [%]	at. [%]	wt. [%]	at. [%]	wt. [%]	at. [%]	wt. [%]	at. [%]	wt. [%]	at. [%]	wt. [%]	at. [%]	wt. [%]
O	3.26	0.96	5.81	1.75	2.96	0.87	2.72	0.80	8.25	2.53	30.32	11.47	3.38	1.00	2.98	0.88
Cr	15.26	14.61	16.89	16.49	18.35	17.57	18.52	17.70	18.04	17.99	40.18	49.40	18.80	18.09	17.70	16.95
Fe	68.72	70.64	64.67	67.83	66.07	67.92	65.97	67.70	64.61	69.23	26.98	35.63	68.75	71.05	69.09	71.10
Ni	12.76	13.79	12.63	13.93	12.62	13.64	12.79	13.80	9.10	10.25	2.52	3.50	9.07	9.86	10.23	11.07

## Data Availability

The data presented in this study are available on request from the corresponding author. The data are not publicly available due to privacy.
